# Non-Triple Helical Form of Type IV Collagen α1 Chain

**DOI:** 10.1016/j.heliyon.2015.e00051

**Published:** 2015-12-09

**Authors:** Hiroaki Sugiyama, Kazuhiro Tokunaka, Toshihiko Hayashi, Yasutada Imamura, Makoto Morita, Masayuki Yamato

**Affiliations:** a Institute of Advanced Biomedical Engineering and Science, Tokyo Women's Medical University, Tokyo 162-8666, Japan; b Pharmaceutical Research Laboratories, Nippon Kayaku Co., Ltd., Tokyo 115-8588, Japan; c China-Japan Research Institute of Medical and Pharmaceutical Sciences, Shenyang Pharmaceutical University, Shenyang 110016, China; d Department of Chemistry and Life Science, School of Advanced Engineering, Kogakuin University, Hachioji, Tokyo 192-0015, Japan

**Keywords:** Biological sciences, Cell biology, Adhesion structures, Pathology in cell biology

## Abstract

Type IV collagen with a triple-helical structure composed of three α chains is a major component of basement membrane. Previously, we reported that non-triple helical form of type IV collagen α1 chain (NTHα1(IV)) was isolated from human placenta and the culture media of human cells. In the present study, we report on the localization of NTH α1(IV) with a monoclonal antibody #370, exclusively reactive for the nascent chain, in the rabbit tissues. The staining was found on the basement membrane of blood vessels, of endomysium, of nerve, and of kidney but not on epithelial basement membrane. In a rabbit angiogenic model, #370 antibody staining was exclusively observed in neovascular tip region of endothelial cells, where no staining with anti-type IV collagen antibody was seen. Distinct localizations suggest that NTHα1(IV) is produced and stably deposited in endothelial cells and the surroundings under physiological conditions with some physiological roles in relation to the dynamics of vascular system.

## 1 Introduction

Collagen is a major component of extracellular matrix. Collagen proteins have a triple-helical structure consisting of three α chains. Type IV collagen is deposited at the boundary between epithelial or endothelial tissue and connective tissue as a major component of basement membrane. Six alpha chains, α1(IV) to α6(IV), are known as members of the type IV collagen family. The type IV collagen composed of two α1(IV) and one α2(IV) chains extensively exists in a mammalian body, while other forms of type IV collagen with chain compositions of α3(IV) α4(IV) α5(IV), and [α5(IV)]_2_ α6(IV) are limited in their localizations (Brinckmann et al., 2005). Translated procollagen chains are subjected to post-translational modifications by enzymes, before they assemble into stable triple-helical structures (Prockop and Kivirikko, 1995; Steinmann et al., 1981; Uitto et al., 1972). The procollagen polypeptides that have failed to form the triple-helical conformation are supposed to be degraded inside the cell through the quality control system or proteasome pathway. However, Engvall et al. reported that non-disulfide-bonded non-triple helical type IV collagen polypeptides were found in culture medium of a mouse teratocarcinoma-derived cell line, using fibronectin affinity chromatography (Engvall et al., 1982). Iwata et al. showed that a short form of α1(IV) collagen existed in bovine lens capsule using monoclonal antibody (JK132) that is reactive for α1(IV) collagen chain at the triple helical domain in denatured form (Iwata et al., 1995). Takahashi et al. detected non-disulfide-bonded and unfolded α1(IV) and α2(IV) chains in the culture media of human fetal lung fibroblasts (TIG-1) (Takahashi et al., 1999). Furthermore, Yoshikawa et al. reported that secretion of non-helical collagen polypeptides correlates with depletion of ascorbic acid in culture media of human cells (Yoshikawa et al., 2001). Kajimura et al. revealed that non-disulfide-bonded non-helical α1(IV) chain existed in human placenta, using the specific affinity for lectin agaricus bisporus agglutinin, which did not react with triple helical type IV collagen (Kajimura et al., 2004). These results provide evidence for the stable production and secretion of non-triple helical form of type IV collagen α1 chain (NTHα1(IV)) in mammalian cells. Recently, we developed the mouse monoclonal antibodies including #370 antibody against NTHα1(IV) purified with JK132-affinity column from the culture medium of human hepatocellular carcinoma cells (HLF) in the absence of ascorbate. One of the antibodies, #370 antibody, recognizes nascent and secreted NTHα1(IV) but not the denatured α1 chain from type IV collagen. In the present study, we here report on the tissue distributions of NTHα1(IV) in rabbit tissues, normal and angiogenic model, in comparison with type IV collagen.

## 2 Results

### 2.1 Distributions of NTHα1(IV) in rabbit tissues

**Fig. 1:**
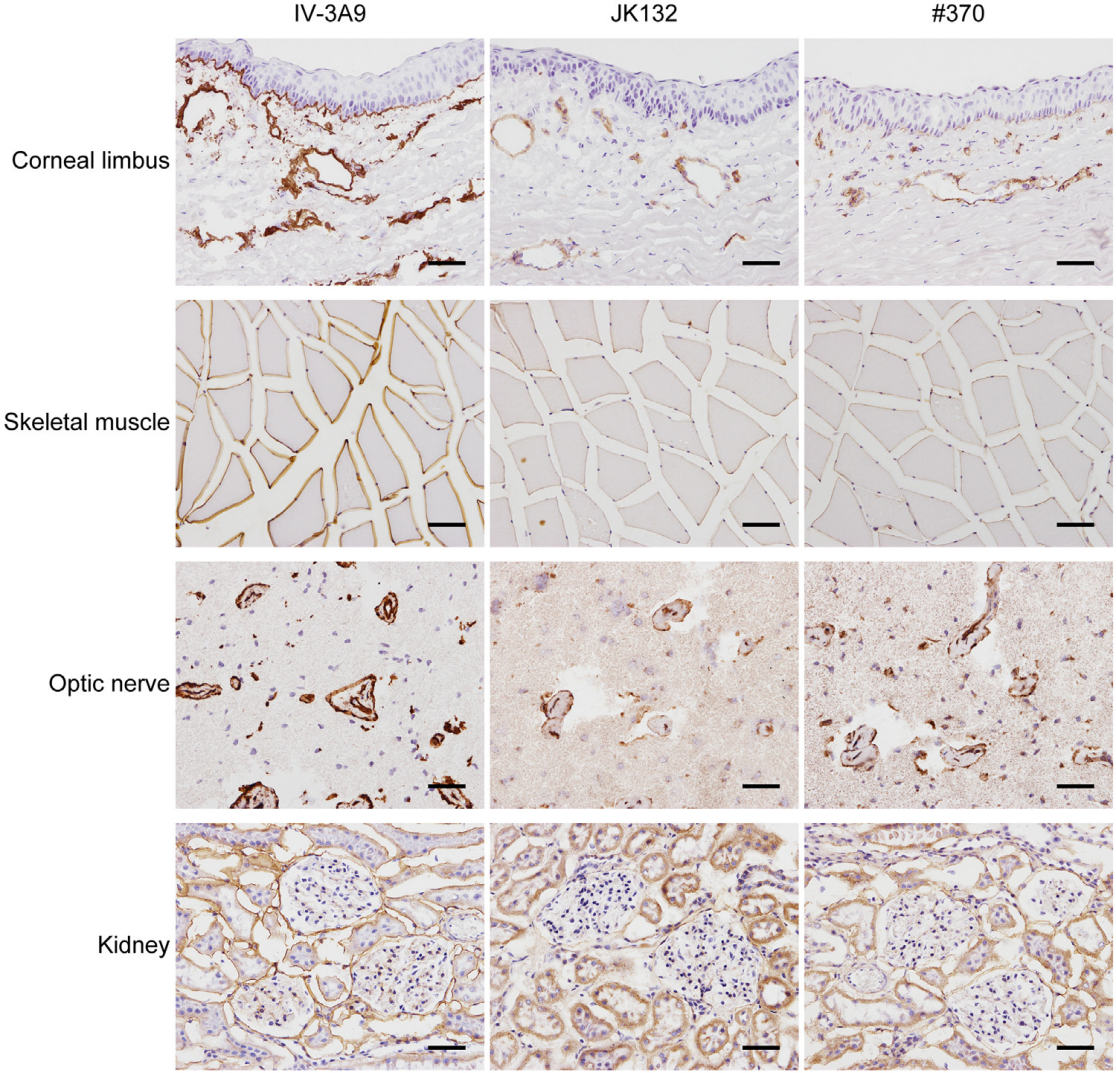
Distributions of NTHα1(IV) in normal rabbit tissues. Normal rabbit corneal limbus, skeletal muscle, optic nerve, and kidney were stained with three antibodies. IV-3A9 antibody staining is observed in epithelial, vessel, muscle, nerve, and kidney (Bowman's capsule and tubular) basement membranes, and the staining is faint in mesangial area of kidney. The stainings with JK132 and #370 antibodies show essentially the same distributions on basement membranes except for the epithelial basement membranes. Scale bar represents 50 μm.

The ocular surface is composed of cornea, conjunctiva and limbus, which is known as a transitional zone between cornea and conjunctiva. Blood vessels are found in the connective tissues under the limbal to conjunctival epithelial layers, but they lack in corneal stromal layer. Type IV collagen components of epithelial basal layer are different between corneal and conjunctival regions. That is, the central region of corneal basement membrane consists of type IV collagens comprising with α3(IV) α4(IV) α5(IV), and [α5(IV)]_2_ α6(IV) chains, and type IV collagen in conjunctival epithelial basement membrane consists of [α1(IV)]_2_ α2(IV) and [α5(IV)]_2_ α6(IV) chains (Guerriero et al., 2007; Kameishi et al., 2015). In the present study, three types of antibodies, IV-3A9, JK132, and #370 antibody, were utilized and these antibodies' epitopes resided within α1(IV) or/and α2(IV) chains. According to the previous works, immunologically positive staining with these antibodies was not expected in corneal epithelium at the central region. Immunologically positive staining with IV-3A9 antibody was obtained in not only conjunctival epithelial basement membrane but also vascular basement membrane, while JK132 and #370 antibody stainings were observed predominantly on vascular basement membrane, but essentially none on epithelial basement membrane (Fig. 1). The basement membranes of endomysium of skeletal muscle tissue and nerve tissue were positively stained with all the three antibodies. In normal rabbit kidney, Bowman's capsule basement membrane and tubular basement membrane were positively stained, while mesangial area was faintly stained with all the three antibodies. Furthermore, the three antibodies also react to human tissues on the basement membranes of blood vessels in normal and tumor tissues (unpublished observations).

### 2.2 Distribution of NTHα1(IV) in angiogenic model in rabbit cornea

**Fig. 2:**
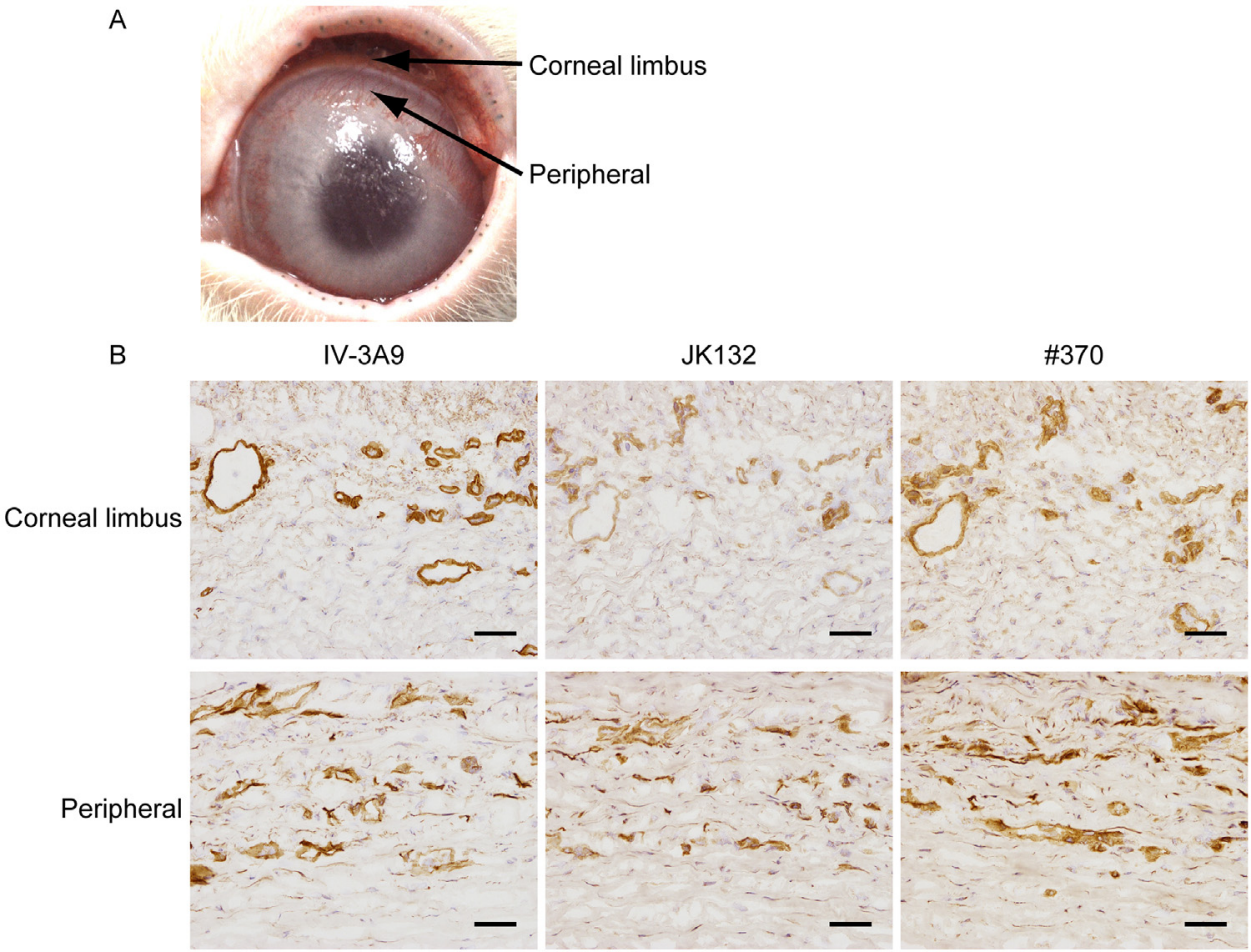
Distributions of the NTHα1(IV) in comparison with type IV collagen in rabbit angiogenic model. (A) Neovascularization became apparent in 2 weeks after surgery. Small vessels are formed in the peripheral region of ocular surface from corneal limbus. Neovascular tissues do not yet cover the entire ocular surface. (B) Ocular surface of rabbit corneal deficiency model was stained with three antibodies. IV-3A9 antibody, anti-type IV collagen antibody, shows staining not only on vascular basement membrane but also epithelial basement membrane. JK132 and #370, anti-NTHα1(IV) antibodies, show predominant stainings on vascular basement membrane, but no staining on epithelial basement membrane. Scale bar represents 50 μm.

When the corneal epithelium is severely damaged, adjacent conjunctival epithelial cells spread into the damaged region and initiates to regenerate the tissue, accompanying neovascularization (Kameishi et al., 2015; Sugiyama et al., 2014; Tseng, 1989). Since cornea has no-blood vessels, the rabbit corneal angiogenic model is one of the useful experimental systems for elucidating the mechanism of neovascularization. Two weeks after corneal damage, neovessels spread into peripheral ocular surface region from corneal limbus; however, neovascular tissue did not cover the entire ocular surface (Fig. 2A). The ocular surface was subjected to immunohistochemical analysis with above-mentioned antibodies. IV-3A9 antibody staining was observed in vascular basement membrane that formed luminal surface form in the connective tissue of corneal limbus region. In peripheral region, IV-3A9 antibody staining was diffuse and random presumably corresponding to immature microvessels (Fig. 2B). JK132 and #370 antibody stainings were also observed in vascular basement membrane of corneal limbal region. Diffuse and random staining was observed in the site of immature microvessels in peripheral region (Fig. 2B).

**Fig. 3:**
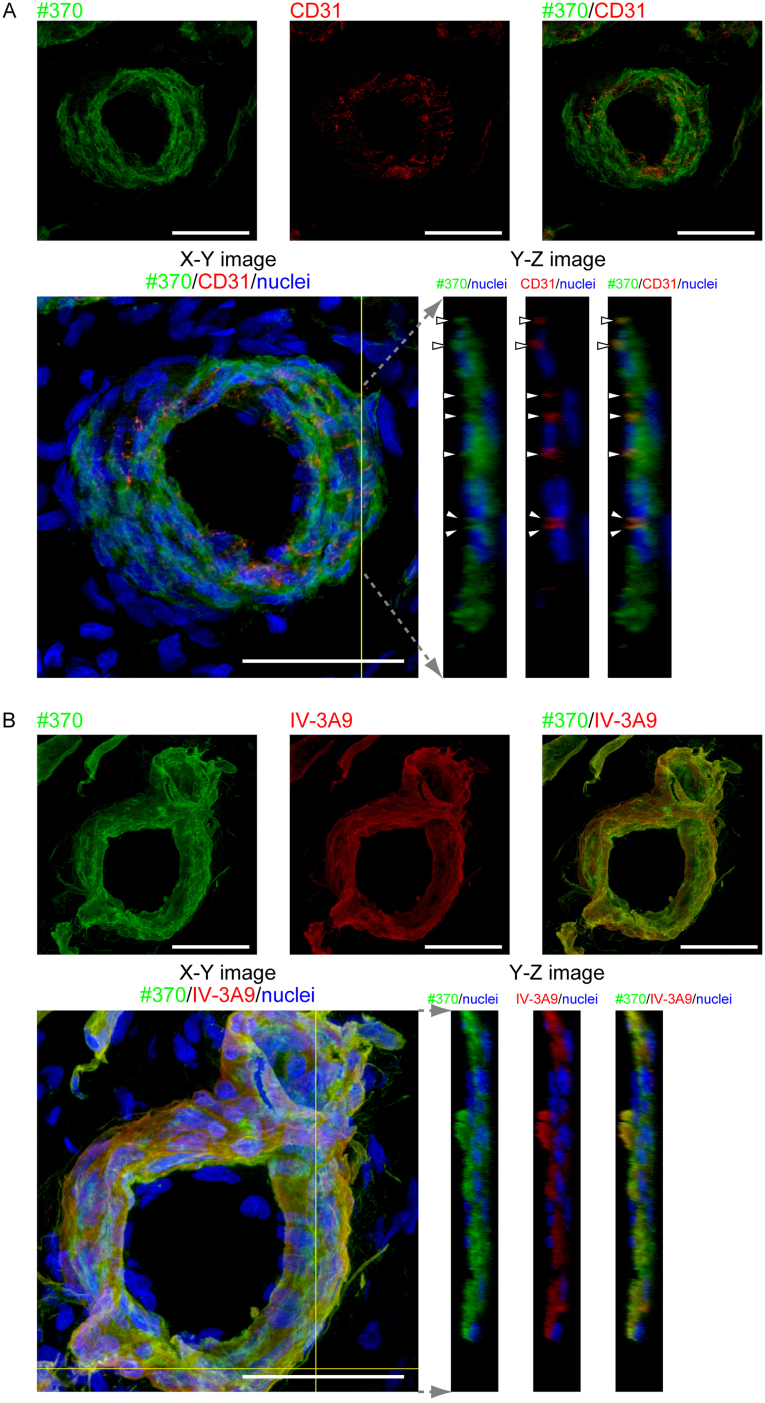
Distributions of type IV collagen and NTHα1(IV) in relation to endothelial cells in rabbit angiogenic model. (A) Ocular surface of rabbit corneal deficient model was stained with #370 antibody and anti-CD31 antibody using the frozen sections with thickness of 25 μm. Cross-section images were obtained with confocal laser scanning microscopy. #370 staining is observed in not only plasma membrane but also cytoplasm in CD31-positive vascular endothelial cells. White arrow heads show the points of CD31 staining representing the plasma membrane of vascular endothelial cells. Scale bar represents 50 μm. (B) Ocular surface of rabbit corneal deficient model was stained with #370 antibody and IV-3A9 antibody. #370 antibody staining colocalizes with IV-3A9 antibody staining in many regions. Scale bar represents 50 μm.

Double immunofluorescent staining was performed to examine the localization of NTHα1(IV) in blood vessels, using frozen sections in 25 μm thickness. CD31 known as platelet endothelial cell adhesion molecule (PECAM-1) is present on the plasma membrane of vascular endothelial cells (Newman et al., 1990). In the blood vessels that have lumina, #370 antibody staining was observed not only on the plasma membrane and proximal region, but also in the cytoplasm of the vascular endothelial cells that were stained with anti-CD31 antibody (Fig. 3A). Furthermore, #370 antibody staining was colocalized with IV-3A9 antibody-positive staining in many regions, indicating that type IV collagen and NTHα1(IV) were deposited at the same or closest region (Fig. 3B).

**Fig. 4:**
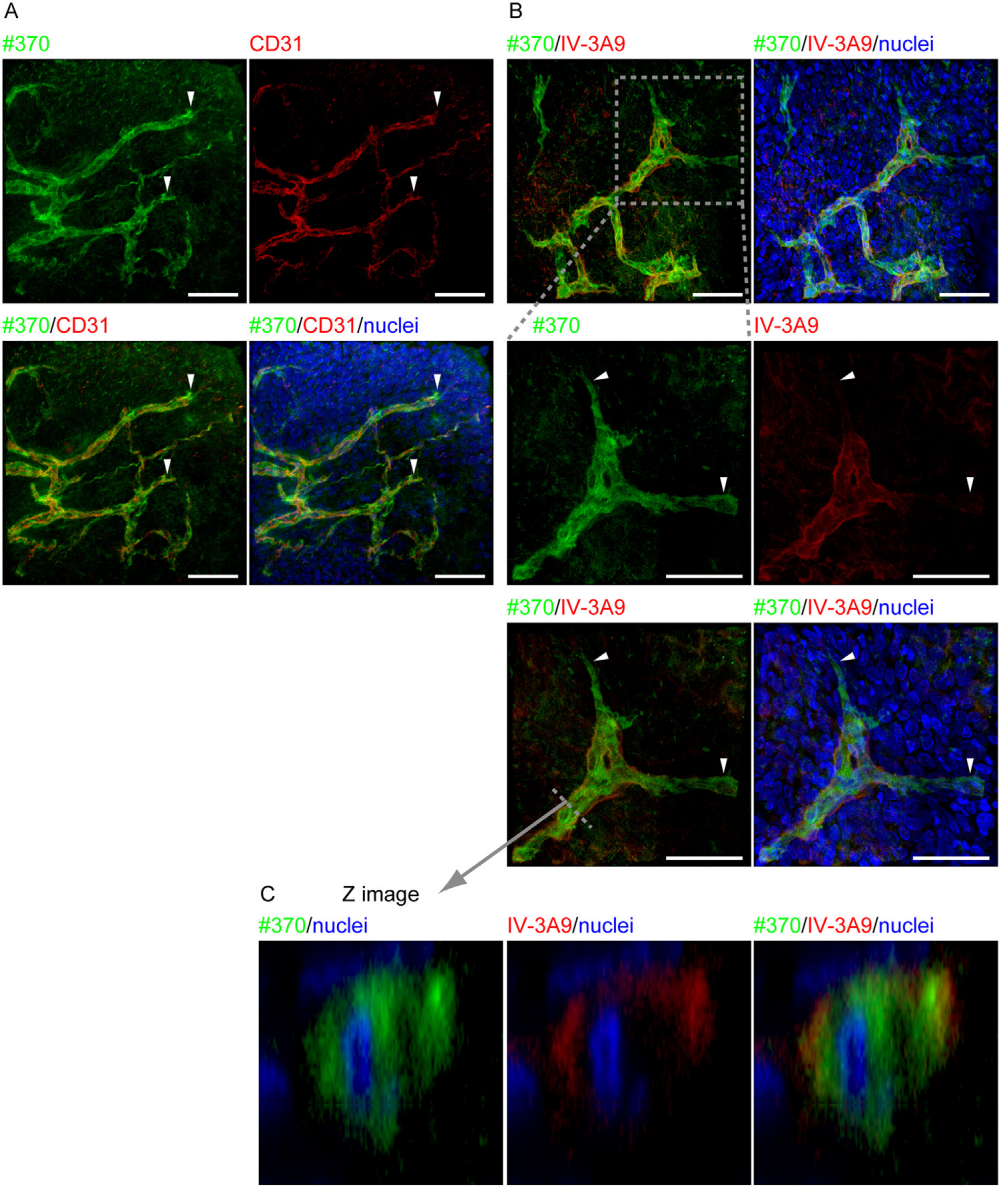
Distributions of type IV collagen and NTHα1(IV) in whole mount specimen (A) #370 antibody staining is observed in CD31 positive vascular endothelial cells at neovascular tip region (white allow heads). Scale bar represents 50 μm. (B) Neovascular tip region is stained with #370 and IV-3A9 antibody. High magnification images are shown (lower panels). The areas stained only with #370 antibody, but not stained with IV-3A9 antibody are discernible at the tip region of neovessels (white arrow heads). Scale bar represents 50 μm. (C) Images of the cross-section at the dashed line were shown. #370 antibody staining is detectable in cytoplasm in addition to the plasma membrane and the cell vicinity, while IV-3A9 antibody staining is detected on cellular lateral sides in the immature neovessels without luminal formation.

Whole mount immunofluorecence staining was performed to examine the tip region of neovessels. CD31 positive vascular endothelial cells were stained with #370 antibody at the tip region of blood vessels (Fig. 4A). On the other hand, the tip region of neovessel where the staining with #370 antibody was marked was not stained with IV-3A9 antibody (Fig. 4B). This is demonstrated more clearly in three dimensional observations by confocal laser scanning microscopy (Supplementary Figure S1). The regions behind the tip region consisting of immature microvessels without luminal formation were stained with both #370 antibody and IV-3A9 antibody. According to imaging analysis, #370 antibody staining was detected in the cytoplasm in addition to the cell surface and vicinity, while IV-3A9 antibody staining was detected only on lateral sides of the cell (Fig. 4C).

## 3 Discussion

Present observations clearly demonstrated that the distributions of NTHα1(IV) in rabbit tissues are distinct from those of the type IV collagen with a chain composition of [α1(IV)]_2_α2(IV) by immunohistological analysis, using the antibody that specifically recognizes nascent NTHα1(IV); predominantly deposited in vascular basement membranes at the tip of premature microvessels where type IV collagen is not detected, but absent in epithelial basement membrane. Distinct localization of NTHα1(IV) implies for distinct functions. Some interactive characteristics of NTHα1(IV) could be relevant to possible functions in vivo. Higher affinity with fibronectin of NTHα1(IV) in comparison with type IV collagen as reported could be important (Engvall et al., 1982).

Ishizaki et al. reported that JK132 antibody staining was detected in vascular basement membrane, ciliary muscle basement membrane, and optic nerve basement membrane, except for limbus to conjunctival basement membrane (Ishizaki et al., 1993). However, limbus to conjunctival-basement membrane was stained after denaturation with urea or pepsin treatment. JK132 antibody prepared by human type IV collagen isolated from placenta as an antigen (Kino et al., 1991) does not react with the type IV collagen in the triple-helical conformation. The sequence in the triple-helical domain of α1(IV) chain, 1165 to 1179 amino-acid, recognized by JK132 antibody is hidden in triple-helical conformation (Iwata et al., 1995; Takahashi et al., 1999). Therefore, epithelial basement membrane was not stained with JK132 antibody without denaturing treatment. JK132 antibody reacts with non-triple helical α1(IV) chain; not only nascent NTHα1(IV) but also the non-triple helical α1(IV) chain derived from triple helical type IV collagen by denaturation. In the present study, we prepared a novel anti-NTHα1(IV) mouse monoclonal antibody, #370, which was prepared by using the NTHα1(IV) purified from human hepatocellular carcinoma cell line (HLF) using JK132 antibody affinity column as the antigen. Accordingly, #370 antibody reacts to human tissues. It has reactivity to rabbit tissues as shown here for the first time. This antibody reacts neither with triple-helical type IV collagen nor with the non-helical α1(IV) chain derived from denaturation of triple-helical type IV collagen (manuscripts on the antibody characterizations in preparation). That is, nascent NTHα1(IV) is so far the only antigen for #370 antibody. In normal rabbit tissues, #370 antibody staining patterns are totally consistent with the JK132 antibody staining patterns without denaturation as reported previously (Hirata et al., 1995; Ishizaki et al., 1993; Makino et al., 1994).

Present immunohistological analyses have provided the evidence that nascent NTHα1(IV) is deposited under physiological conditions. It was previously reported that NTHα1(IV) contained lectin ABA reactivity or an additional glycosylation that the α1(IV) chain from type IV collagen did not have. The present results that preferential localization of NTHα1(IV) at the tip of premature microvessels together with the colocalization with type IV collagen in other microvessels suggest us to hypothesize that NTHα1(IV) could have a key role for angiogenesis and/or homeostasis of vascular system.

Collagen has posttranslational modifications such as prolyl (3- and 4-) hydroxylation. It is generally accepted that prolyl 4-hydroxylation contributes to stability of triple helical structure of collagen (Brinckmann et al., 2005; Vranka et al., 2004). This is consistent with the type IV collagen gene products, with approximately 70% 4-hydroxyproline in triple-helical type IV collage and only 14% hydroxyproline in the NTHα1(IV) (Yoshikawa et al., 2001). A most recent study indicated that embryonic lethal prolyl 3-hydorxylase 2-null mouse is completely rescued by producing double knock out of platelet-specific glycoprotein VI (GPVI), collagen receptor in platelets (Pokidysheva et al., 2014). The report discussed that in prolyl 3-hydroxylase 2-null mouse, maternal platelets aggregated with non-prolyl 3-hidroxylated type IV collagen, and cause death of the embryo by thrombosis. We have found that NTHα1(IV) has an ability to aggregate platelets (unpublished data). Exclusive staining with #370 antibody was observed at the tip of neovessel at advancing area in rabbit angiogenic model, while co-staining with IV-3A9 antibody was observed at the tube formed neovessels (Fig. 4B). NTHα1(IV) might be involved in the restoration of blood vessels and neovascularization by blocking of blood leakage through the platelet aggregation, especially at the tip region of neovessels.

Microvessels consist of endothelial cells and pericytes on the abluminal surface. Basement membrane exists between endothels and pericytes (Armulik et al., 2005). It was reported that pericytes produced the type IV collagen and laminin that consist of vascular basement membrane (Sundberg et al., 1996). NTHα1(IV) is secreted without intracellular degradation in different kinds of human cells including fetal lung fibroblasts (TIG-1), mesangial cells (HMC), umbilical vascular endothelial cells (HUVEC), aortic smooth muscle cells (HASMC), etc. NTHα1(IV) can be isolated from human placenta (Kajimura et al., 2004). Therefore, production of NTHα1(IV) by pericytes cannot be excluded, even though double immunofluorescence staining with CD31, IV-3A9, and #370 antibody suggested that type IV collagen and NTHα1(IV) were deposited at direct vicinity of endothelial cells. In the neovascular tissue, #370 and JK132 antibody staining was observed in not only endothelial vicinity but also the places distant from endothelial cells that are in a scattered way (Fig. 2). Since the promotion of neovascularization by pericyte in wound healing has been suggested (Morikawa and Ezaki, 2011), we presume that NTHα1(IV) is secreted not only by endothelial cells but also by pericytes for neovascularization.

Another monoclonal antibody was prepared, by using thermally denatured pepsin treated human type IV collagen as an antigen (Xu et al., 2001). This antibody predominantly reacts to denatured/proteolyzed type IV collagen, show little reactivity to triple-helical type IV collagen. This antibody inhibited angiogenesis and tumor growth (Xu et al., 2001). NTHα1(IV) may well react with the antibody. Neovascularization is thought to be essential for the progression of tumors. Thus, NTHα(IV) and other NTHα chains could be a potential novel target(s) for malignant tumor therapy. Furthermore, lysyl oxidase secreted from hypoxic tumor cells mediates the formation of premetastatic niche, accumulates at premetastatic site, and provides crosslinking of type IV collagen in the basement membrane, resulting in recruitments for myeloid cells. These myeloid cells adhere to crosslinked collagen, produce matrix metalloproteinase-2, which cleaves basement membrane, and enhance the tumor cell metastasis (Erler et al., 2009). NTHα1(IV) is secreted by mouse teratocarcinoma-derived cell line (PF HR-9) (Engvall et al., 1982), human fibrosarcoma cell HT-1080 (Kajimura et al., 2004) and other carcinoma cell lines(unpublished data). We assume that NTHα1(IV) might contribute to formation of niches; for premetastatic cells on the one hand, for somatic stem cells on the other hand. Niches are considered to be maintained within appropriate environments including cell-cell interactions, extracellular matrix components, growth factors, cytokines and so on. Previously we reported that hematopoetic stem cells (Umemoto et al., 2012) and corneal limbal stem cells (Kusanagi et al., 2009) express integrin β3 that forms heterodimers with integrin αIIb and αv (Barczyk et al., 2010). Integrin αvβ3 has binding ability to denatured type IV collagen, but not to triple-helical type IV collagen (Xu et al., 2001). The observations suggested us to assume that NTHα(IV) chains can be one of the stem cell niche factors.

The present finding that NTHα1(IV) is exclusively expressed in the tip region of neovessels lead us to hypothesize that angiogenesis or proliferation and migration of endothelial cells is regulated in part by NTHα1(IV).

## 4 Materials and methods

### 4.1 Preparation of anti-NTHα1(IV) antibody (#370 antibody)

NTHα1(IV) was purified from culture medium of human hepatocellular carcinoma cell line, HLF (Riken Cell Bank, Japan), using JK132 monoclonal antibody-coupled affinity column. The spleen cells were obtained from Balb/c mice immunized with NTHα1(IV) and then hybridoma cells were prepared. #370 antibody was isolated from one type of hybridoma cells. For double immunofluorescence staining, #370 antibody was biotinylated using Micro Sulfo-NHS-LC-Biotinylation Kit (Thermo Fisher, MA, USA). Slufo-NHS-LC-biotin solution (3.7 μl, 9 mM) was add to the antibody solution (200 μl, 500 μg/ml), and incubated at 4 °C for overnight. The biotinylated antibody solution was purified by spin desalting column to remove excess biotin, and stored at 4 °C until use.

### 4.2 Angiogenic model in rabbit cornea

New Zealand White rabbit was treated in accordance with an experimental procedure approved by the Animal Care and Use Committee at Tokyo Women's Medical University. To fabricate an angiogenic model, the corneal defect was created by surgically removing of epithelium including limbus and conjunctival tissue and exposing the stroma. After keratectomy, the ocular surface was treated with the topical application of 1-*n*-heptanol for 5 minutes. A few drops of an antibiotic (0.3% ofloxaxin) (Santen, Osaka, Japan) and steroid (0.1% betamethasone) (Shionogi, Osaka, Japan) were put once a day for 1 week. Two weeks after surgery, the rabbit was sacrificed and subjected to immunohistological examinations.

### 4.3 Immunohistological analysis

For immunohistochemical analysis, rabbit cornea, kidney, muscle, and optic nerve were embedded in an optimum cutting temperature compound and processed into 5 μm frozen sections. Tissue sections were treated with three kinds of antibodies, IV-3A9 (Daiichi Fine Chemical, Toyama, Japan), JK132, and #370. Secondary antibody was horseradish peroxidase (HRP) conjugated anti-mouse IgG (Jackson immune Research Laboratories, West Grove, PA). The sections were stained by the treatment with 3,3′-diaminobenzidine tetrahydrochloride and counterstained with hematoxylin. For double immunofluorescence staining, frozen sections were prepared into 25 μm thickness and whole mount rabbit corneal tissues were fixed with 70% ethanol. These specimens were stained with mouse monoclonal antibodies, anti-CD31 (Dako, Glostrup, Denmark) or IV-3A9 were detected by the treatment with Alexa 594 conjugated anti-mouse IgG (Jackson Immuno Research Laboratories), biotin conjugated #370 was detected by the treatment with Alexa 488 conjugated streptavidin (Jackson Immuno Research Laboratories), and observed with a confocal laser scanning microscope (LSM-510) (Carl Zeiss, Oberkochen, Germany).

## Declarations

### Author contribution statement

Hiroaki Sugiyama: Conceived and designed the experiments; performed the experiments; wrote the paper.

Kazuhiro Tokunaka: Performed the experiments.

Makoto Morita: Conceived and designed the experiments; performed the experiments; analyzed and interpreted the data.

Toshihiko Hayashi, Yasutada Imamura and Masayuki Yamato: Conceived and designed the experiments; performed the experiments; analyzed and interpreted the data; wrote the paper.

### Funding statement

This work was supported by the Formation of Innovation Center for Fusion of Advanced Technologies in the Special Coordination Funds for Promoting Science and Technology “Cell Sheet Tissue Engineering Center”; the Global COE program; and the Multidisciplinary Education and Research Center for Regenerative Medicine from the Ministry of Education, Culture, Sports, Science and Technology, Japan.

### Competing interest statement

The authors declare no conflict of interest.

### Additional information

No additional information is available for this paper.

## Supplementary content

Fig. S1 Three-dimensional movie of whole mount immunofluorescent staining. Three-dimensional image of the tip region was created by IMARIS software. Staining of a tip region of neovessel is confirmed by three-dimensional imaging analysis. The region is stained with only #370 antibody, but not stained with IV-3A9 antibody.
