# Supplementary figures and images for: Non-Triple Helical Form of Type IV Collagen α1 Chain
Source: Heliyon. 2015 Dec 9;1(4):e00051. doi: 10.1016/j.heliyon.2015.e00051 (PMC4945737; doi:10.1016/j.heliyon.2015.e00051)

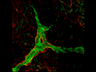

Supplement: Fig. S1 — Three-dimensional movie of whole mount immunofluorescent staining. Three-dimensional image of the tip region was created by IMARIS software. Staining of a tip region of neovessel is confirmed by three-dimensional imaging analysis. The region is stained with only #370 antibody, but not stained with IV-3A9 antibody. [file mmc1.jpg]
